# Image-based time series analysis to establish differential disease progression for two *Fusarium* head blight pathogens in oat spikelets with variable resistance

**DOI:** 10.3389/fpls.2023.1126717

**Published:** 2023-03-14

**Authors:** Mirko Pavicic, Katriina Mouhu, Juho Hautsalo, Daniel Jacobson, Marja Jalli, Kristiina Himanen

**Affiliations:** ^1^ National Plant Phenotyping Infrastructure, Helsinki Institute of Life Science (HiLIFE), University of Helsinki, Helsinki, Finland; ^2^ Department of Agricultural Sciences, Viikki Plant Science Centre, Helsinki, Finland; ^3^ Computational and Predictive Biology, Biosciences Division, Oak Ridge National Laboratory, Oak Ridge, TN, United States; ^4^ Natural Resources Institute Finland (Luke), Management and Production of Renewable Resources Planta, Jokioinen, Finland; ^5^ Bredesen Center, University of Tennessee Knoxville, Knoxville, TN, United States; ^6^ Organismal and Evolutionary Biology Research Programme, Biocenter Finland, University of Helsinki, Helsinki, Finland

**Keywords:** *Fusarium* head blight, cereals, image based phenotyping, chlorophyll fluorescence, oats, time series analysis

## Abstract

Oat-based value-added products have increased their value as healthy foodstuff. *Fusarium* head blight (FHB) infections and the mycotoxins accumulated to the oat seeds, however, pose a challenge to oat production. The FHB infections are predicted to become more prevalent in the future changing climates and under more limited use of fungicides. Both these factors increase the pressure for breeding new resistant cultivars. Until now, however, genetic links in oats against FHB infection have been difficult to identify. Therefore, there is a great need for more effective breeding efforts, including improved phenotyping methods allowing time series analysis and the identification of molecular markers during disease progression. To these ends, dissected spikelets of several oat genotypes with different resistance profiles were studied by image-based methods during disease progression by *Fusarium culmorum* or *F. langsethiae* species. The chlorophyll fluorescence of each pixel in the spikelets was recorded after inoculation by the two *Fusarium* spp., and the progression of the infections was analyzed by calculating the mean maximum quantum yield of PSII (F_v_/F_m_) values for each spikelet. The recorded values were (i) the change in the photosynthetically active area of the spikelet as percentage of its initial size, and (ii) the mean of F_v_/F_m_ values of all fluorescent pixels per spikelet post inoculation, both indicative of the progression of the FHB disease. The disease progression was successfully monitored, and different stages of the infection could be defined along the time series. The data also confirmed the differential rate of disease progression by the two FHB causal agents. In addition, oat varieties with variable responses to the infections were indicated.

## 1 Introduction

Mycotoxin-producing *Fusarium* species cause severe *Fusarium* head blight (FHB) infections in cereals. FHB is a global problem that can be associated with major yield losses and considerable threat of mycotoxin contamination of wheat and barley both in North America and in Europe (McMullen et al., 1997; Goswami and Kistler, 2004; Johns et al., 2022). In Nordic countries, the most severely influenced cereal is oats, an important crop for providing food, feed, and export income. In recent years, there have been several large investments in the oat-derived food processing industry. The production of high-quality oat grain is, however, compromised by FHB infections, which can reduce grain yield directly (Kiecana et al., 2002) through death of developing grains, due to severe infections (Tekle et al., 2013), or indirectly by making the grains unacceptable for food use by accumulating mycotoxins into the grain (Bjørnstad and Skinnes, 2008). FHB is a challenging disease to manage, because it has multiple causal agents with different environmental preferences and the different *Fusarium* spp. can cause disease in several host species. For example, deoxynivalenol (DON) mycotoxin is produced mainly by *Fusarium graminearum* (Schwabe, 1839) in Nordic countries (Hietaniemi et al., 2016; Hofgaard et al., 2016), but in cool growing seasons, *F. culmorum* (Wm.G. Sm.) Sacc. Syll. fung. (Abellini) 10: 726. (1892) may become the main DON producer (Kaukoranta et al., 2019). Additionally, T-2/HT-2 mycotoxin is produced by *Fusarium* species such as *F. langsethiae* (Torp and Nirenberg, 2004) and *F. sporotrichioides* (Sherb, 1915), which can dominate on years associated with warm and dry weather after anthesis (Kaukoranta et al., 2019). The further we develop our knowledge of the different mycotoxin risks associated with different *Fusarium* fungi in cereals, the higher is also the pressure towards tightening the regulative legislation of mycotoxin contents of food products (Johns et al., 2022).

The resistance of oats to both DON (Tekle et al., 2018) and T-2/HT-2-producing fungi (Isidro-Sánchez et al., 2020) can be assessed in artificially inoculated field trials but several trials are needed to get consistent cultivar rankings (Tekle et al., 2018; Hautsalo et al., 2020). Controlled environments can provide more consistent estimates for the disease, but these results do not necessarily fit for selecting cultivars that would have resistance in variable field conditions (Hautsalo et al., 2020) due to the complex nature of FHB disease resistance. For example, FHB symptoms can be quantified, but they correlate weakly with DON mycotoxin contents in oats, whereas germination and DON levels (Tekle et al., 2018) or *F. graminearum* DNA levels and DON levels show better correlations (Yli-Mattila et al., 2009; Yli-Mattila et al., 2017). In field conditions, the host plants can, however, avoid the disease due to their morphological and phenological features such as flowering time or habit (Herrmann et al., 2020) and the progress and severity of the disease can also vary depending on the weather conditions. Until and even after genomic prediction models are developed for selecting resistance in breeding material, expensive mycotoxin testing must be performed in large scale. Taking into account the complexity of the disease progression, a better understanding of the development of the disease is required to determine the phenotypes indicative of the spatial and temporal resistance factors.

*F. graminearum, F. culmorum*, and *F. langsethiae* infect wheat and barley spikes and oat panicles during anthesis (Schroeder et al., 1963; Wagacha and Muthomi, 2007; Tekle et al., 2012; Divon et al., 2019). *Fusarium* spores are more likely to land on outer floral parts than inside the florets of oats (Tekle et al., 2012; Xue et al., 2015) and thus they must grow hyphae towards the soft floral interior tissue. In inner surfaces of the flowers, they can form infection structures such as infection cushions and foot-like structures (appressoria, Boenisch and Schäfer, 2011; Divon et al., 2019), which can penetrate cell walls and produce mycotoxins. In order to measure the infection and oat responses at a spikelet level in oats, Hautsalo et al. (2020) applied a point inoculation method to infect *F. graminearum* into oat spikelets. However, the two oat genotypes investigated that show large differences in their DON accumulation and proportion of *Fusarium*-infected kernels in greenhouse experiments did not differ in their fungal biomass accumulation. This suggests that if the infection is established inside the floret, there might be no mechanism preventing the spread of the fungus in oats. Typical symptoms in the field are spikelets that have lost their chlorophyll and are covered by mycelium, but this is only an end point result and a method that would allow following disease progression over time would increase our understanding of the phenomenon.

The changes during *Fusarium* infection at the cellular level are similar but slower in resistant plants compared to susceptible plants (Kang and Buchenauer, 2000), and the resistance mechanisms against the initial infection and infection spread are rather similar in wheat (Walter et al., 2010). There are several established methods for controlled monitoring of the disease progression and severity of FHB in cereal. For example, Imathiu et al. (2009) screened detached wheat and oat leaves inoculated with a drop of *F. langsethiae* suspension and observed visible symptom development within 7 days. Bauriegel et al. (2011b) inoculated wheat spikes with *F. culmorum* and observed the cumulative F_v_/F_m_ over time. Tan et al. (2020) used both detached leaf assays and inoculation of wheat spikes to observe their interaction of two co-inoculated *Fusarium* species and received similar results from both infection systems. In contrast to FHB disease in wheat, however, the symptoms of *F. langsethiae* in oats are especially difficult to observe (Divon et al., 2012) and FHB symptoms are not a reliable way of producing oat cultivar ranking in FHB disease resistance (Hautsalo et al., 2018). Moreover, the oat panicle creates a physical obstacle that, in contrast to wheat, efficiently restricts the spread of the pathogens from a spikelet to another (Langevin et al., 2004). This leads to a situation where an inoculated panicle is likely to contain both infected and healthy spikelets that are scattered randomly. Therefore, the detached leaf assays or monitoring of inoculated panicles is not optimal to describe the disease progression at the organ level, and an approach focusing on the spikelets is needed.

Image-based phenotyping offers a solution for a non-invasive monitoring of cellular changes that occur during host–pathogen interaction (Berger et al., 2007). Non-destructive imaging, such as fluorescence and hyperspectral imaging, is already being applied to screen for *Fusarium* resistance of wheat spikes (Bauriegel et al., 2011a). We therefore set out to develop an oat spikelet infection system using a stationary imaging facility to allow monitoring the disease progression by time series of red, green, and blue (RGB) and chlorophyll fluorescence (ChlFl) imaging. The aim was to bring the FHB infection into a controlled condition where the disease progression could be imaged in time series by multiple sensors. Placing the dissected, infected spikelets on petri plates allowed their frequent imaging and thereby analysis of the disease progression in individual spikelets. In this study, we also developed an automated pipeline for pixel-specific ChlFl data extraction tailored for the analysis of the infected spikelet. This approach allowed recording temporal and spatial differences between *Fusarium* species in their rate of disease progression and severity, as well as the response time of oat genotypes. In the future, the established time series analysis will hopefully pave the way for targeted molecular omics assessment of the molecular mechanisms of oat resistance.

## 2 Materials and methods

### 2.1 Plant material

Oat genotypes with known variation in their resistance against *Fusarium* spp. were selected for the experiments with *F. langsethiae* and *F. culmorum* infections. A commercially rejected oat variety, BOR31 (Boreal Plant Breeding Ltd., Finland), that is known to be highly susceptible towards DON accumulation and grain infection in spray inoculated greenhouse experiments (Hautsalo et al., 2020) was used as a susceptible control in both experiments. For the *F. langsethiae* infections, the cultivar Vinger from Graminor Ltd. (Norway) was used as resistant candidate, together with Akseli, Belinda, Odal, BOR01, BOR02, and BOR31. For *F. culmorum* infections, two breeding lines (BOR01 and BOR02) that had shown potential resistance against FHB in oats in previous experiments (Hautsalo et al., 2020) were infected together with BOR31. Each oat genotype was grown in five replicates and the experiment was replicated twice over time. Sowing of the two experiments was adjusted according to the oat growing time in greenhouse so that flowering spikelets from each genotype could be harvested at the same time for the two batches of the inoculation experiments. Thus, the experiment was performed twice at two different times during autumn 2019 and winter 2020. We refer to each repetition as batch 1 and 2 in the rest of the manuscript.

### 2.2 Growth conditions at NaPPI facilities

**Figure 1:**
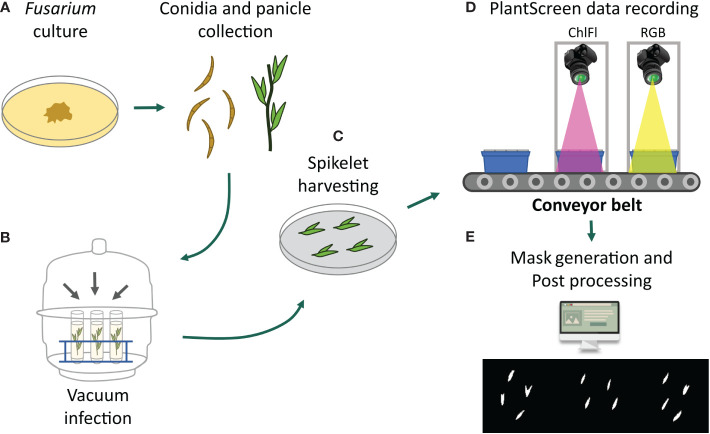
Collage of the experimental workflow. **(A)** *Fusarium* cultures were prepared for conidia collection at the same time when the oat panicles were getting ready for inoculation. **(B)** Vacuum inoculation of panicles was performed within an exicator. **(C)** Dissected spikelets were placed on agar plates in exactly defined coordinates for automated detection by FIJI. **(D)** Imaging of spikelets on the petri plates placed on the blue imaging trays by RGB or ChlFl imaging. **(E)** Detection of mycelia growth symptoms by visible observations on RGB images and by ChlFl pixel data extraction for recording disease progression and severity.

The selected oat genotypes were sown in 3.5-L rose pots (Sparco, Conde-sur-Huisne, France) filled with Kekkilä OPM 420 W R8045–NPK 15-5-24 (Vantaa, Finland) and grown at the Modular plant phenotyping unit of the National Plant Phenotyping infrastructure (NaPPI, https://www2.helsinki.fi/en/infrastructures/national-plant-phenotyping) greenhouse facility at the University of Helsinki, Finland. The day/night temperature was set at 19/13°C and the day length was 18 h supplemented with high-pressure sodium lamps (OSRAM GmbH, Munchen, Germany) regulated at 200 W/m^2^. After the first week of growth, seedlings were fertigated with Kekkilä Professional Vihannes-Superex (NPK 9-5-31, 1.5 mS > 1.2 mS > 1.0 mS) with progressively reducing mS till the plants reached flowering stage and spikelets were ready for dissecting for the *Fusarium* inoculation experiments (**Figure 1**).

For *in vitro* spikelet infection assays, the PlantScreen™ Compact System for small plant imaging was used. The imaging unit is in a controlled environment FytoScope Walk-In chamber (Photon System Instruments, PSI, Czech Republic; www.psi.cz) and accommodates up to 18 imaging trays that are transported on conveyor belts between the visible light (VIS-RGB) imaging cabinet, the ChlFl imaging cabinet, and the light-isolated acclimation chamber. The growth conditions were set at photosynthetically active radiation (PAR) from 150 μmol m^−2^ s^−1^, 18/6 h day length, RH 60%, and temperature 22°C. As the sterile growth media, plant agar (Duchefa Biochemie bv, Haarlem, The Netherlands) with ½ strength Murashige and Skoog media (MS, Duchefa Biochemie bv, Haarlem, The Netherlands) was used for spikelet inoculations and maintenance. The media were autoclaved and applied on round 9-cm petri plates.

### 2.3 Fungal material

All fungal isolates used in the experiments were collected from Finnish cereal fields and were shown to produce mycotoxins in previous studies, *F. culmorum* (isolate 05015) (Kokkonen et al., 2010) and *F. langsethiae* (isolate 05010) (Kokkonen and others, 2011). The isolates were grown as single mycelium cultures on potato dextrose agar (PDA) at 18°C in the dark (**Figure 1A**). After 1 week, the spore production was initiated by culturing it under ultraviolet (UV-A) light (wavelength 350–400 nm, 12 h per day) for 2 weeks. The cultures ready to be used for inoculation were stored at 4°C. Tween-80 (0.01%) was added to inoculation suspensions to obtain uniform dispersion of conidia (**Figure 1A**).

### 2.4 Inoculation of oat spikelets

The inoculum of *F. langsethiae* and *F. culmorum* was applied by submerging the panicles in conidia suspension under vacuum treatment (**Figure 1B**). For vacuum inoculation, one flowering panicle from five separate plants per genotype were harvested into 50-ml tubes. The *Fusarium* spp. inoculums (0.5 × 10^5^ conidia/ml) were added in the tube, and the tubes were placed in an exicator (**Figure 1**). In addition, a control treatment with only water was included. After vacuum treatment of 30 min, the panicles were briefly washed with water. Four spikelets from each of the five individual panicles were dissected and placed on petri plates, resulting in 20 spikelets per genotype treatment and altogether 40 spikelets from the two replicate batches. The position and orientation of all spikelets on the plates were kept fixed to later allow their automated detection (**Figure 1C**). The plates were randomized on blue imaging trays, 9 plates per tray. Trays were kept in an imaging chamber, and their lids were opened just before imaging (**Figure 1D**). Imaging was done at 12- or 24-h interval and continued until 7–9 days, except for the *F. culmorum* where the imaging ended at 3–5 days, at the point when mycelia overgrowth was complete.

### 2.5 Visible light RGB and chlorophyll fluorescence imaging

To assess the progression of *Fusarium* spp. mycelia growth and changes in spikelet appearance, indicating the disease progression, top view images were taken with visible light RGB and ChlFl FluorCam sensors as described in Pavicic et al (2017; 2019; 2021). Images of the petri plates were taken every 12–24 h. Top view RGB2 images (resolution 2,560 × 1,920 pixels) of individual trays were captured with GigE uEye UI-5580SEC/M 5 Mpx Camera (IDS, Germany) with SV-0814H lens, supplemented with an LED-based light source to ensure homogeneous illumination of the imaged object. Light conditions, petri plate positions on imaging trays and spikelet positions on plates, and camera settings were fixed throughout the experiments. Lids of the petri plates were removed before imaging.

The ChlFl measurements were performed with a FluorCam FC-800MF pulse amplitude modulated (PAM) chlorophyll fluorometer as described in Pavicic et al. (2021). The ChlFl illumination panel (FluorCam SN-FC800-195) has pulse-modulated short-duration red–orange flashes (620 nm), a red–orange actinic light (620 nm) with a maximum photosynthetic photon flux density (PPFD) of 300 μmol m^−2^ s^−1^, a cool white actinic light with a maximum PPDF of 500 μmol m^−2^ s^−1^, and a saturating light pulse with a maximum PPDF of 3,000 μmol m^−2^ s^−1^. The ChlFl imaging was done according to the principal F_v_/F_m_ protocol (Tschiersch et al., 2017) that generated parameter images of minimum fluorescence (Fo) and maximum fluorescence (Fm) yields (Murchie et al., 2013). To score the spikelet vitality, a common plant stress indicator, the quantum yield of photosystem II (F_v_/F_m_) was utilized (Barbagallo et al., 2003; Berger et al., 2007). The light intensities were set at Act2 30%, and super 50%, and the FluorCam protocol included 20 min of dark adaptation.

### 2.6 Image processing and image analysis

In VIS-RGB images, the spikelets were observed individually, and observations were recorded according to their coordinates on the plates. RGB images were observed for visible changes in mycelia growth and spikelet appearance such as color. Changes in these parameters were used as indicators of the disease progression. Ranking of the disease severity was based on visible damage and the disease progression was established as a range of spikelet coverage with mycelia. The coverage was ranked as percentage of the whole spikelet (0%, 25%, 50%, 75%, 100%) and other damage (yellowing, dark spots). The ChlFl image pre-processing, automated object detection, and recording of the ChlFl signal were performed using Fiji Is Just ImageJ (FIJI) software version 1.53 (Schindelin et al., 2012; Rueden et al., 2017) using a modified version of the method described in Pavicic et al. (2021). First, raw ChlFl images in.fimg format were imported to FIJI as image type = 32-bit Real, width = 720, height = 560, offset = 8, and little-endian byte order, and saved as.tiff to ease the following pre-processing. Spikelet masks were created from F_o_ images by a simple gray pixel value thresholding of 50 to create a selection of all spikelet pixels. This value was enough to capture whole spikelets while avoiding background pixels. The FIJI function Convert to Mask was used to convert the thresholded pixels to a binary image with background pixel values of 0 and spikelet pixel values of 255. Mask images were used to create a selection of background pixels only (**Figure 1E**). The selection was then transferred to F_v_/F_m_ images and background pixel values were set to –100 for easy filtering in the data wrangling stage. Masked F_v_/F_m_ images were saved as.tiff format. Finally, a rectangle was drawn around each spikelet using their pixel coordinates and the F_v_/F_m_ value for all pixels within the rectangle was saved as Comma Separated Values (CSV) format using the FIJI function Save XY coordinates. All these steps are contained in four modular scripts using FIJI internal macro language (https://github.com/mipavici/MDPI_leaf_infection/).

### 2.7 Data analysis

The output CSV files contain pixel intensities, and X and Y pixel coordinates in the image per spikelet per time point. The CSV files were processed with R programming language in Jupyter Lab using dplyr and tidyr packages (Kluyver et al., 2016; Wickham and Girlich, 2022; Wickham et al., 2022). CSV filenames contained information about the experiment, tray identification, and spikelet position. Thus, they were imported and added to the initial table. All background pixels were removed by keeping F_v_/F_m_ > −100, and cultivar and treatment information were combined with the table. In some cases, at the edge of the spikelet or in late hours post inoculation (HPI) where *Fusarium* spp. mycelia covered the spikelet fluorescent tissue, some F_o_ values can be higher than F_m_, creating a very low negative F_v_/F_m_. To account for this, all negative F_v_/F_m_ were set to 0. Two phenotypes were created to assess infection progression: fluorescent area of spikelets in percentage and spikelet vitality. Fluorescent area of spikelets was calculated by counting the number of fluorescent pixels at each HPI and dividing it by the total fluorescent pixel count at 0 HPI. Spikelet vitality was calculated by averaging the F_v_/F_m_ values of all fluorescent pixels at each HPI. Some plates were removed from the analysis after they were completely covered with mycelia. When this happened, fluorescent area of spikelets in percentage and spikelet vitality were set to 0 for the rest of the time course.

### 2.8 Exclusion criteria from the analysis

Some spikelets opened and closed after transferring them to MS media on petri plates. This created an artificial increment of spikelet size. To account for this artifact, any spikelet that exceeded 5% of its initial size at any point during the time course was completely removed from the analysis. Furthermore, uninfected and dried spikelets were removed from the analysis. From a total of 680 spikelets, 49 and 94 spikelets were removed from batch 1 and 2, respectively.

### 2.9 Statistical analysis of the data

To analyze the infection progression curves, the area under the disease progression curve (AUDPC) of both disease phenotypes for each spikelet was calculated according to Simko and Piepho (2012). In this way, one value per time course was obtained. Linear mixed models were used to assess differences between AUDPCs of cultivars in each treatment. The AUDPCs were modeled using the *lme4* R package (Bates et al., 2015) using cultivars as the main effect and batches were fitted as random effect. Tukey Honestly Significant Difference (HSD) was calculated as *post-hoc* test using the *multcomp* R package (Hothorn et al., 2008). Summaries of statistical analysis and raw data used to create figures are provided as **Supplementary Tables 1**-**4**.

## 3 Results

**Figure 2:**
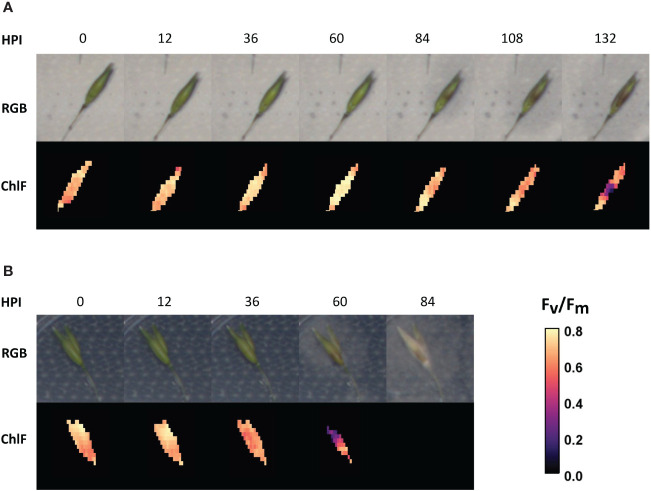
Time series of oat genotype BOR31 spikelets after inoculation with the two *Fusarium* species. **(A)** The progression of *F. langsethiae* hyphae growth over an example spikelet (RGB) and the decline of ChlFl in the same sample. **(B)** The progression of *F. culmorum* hyphae growth on one BOR31 spikelet by RGB and the decline of chlorophyll fluorescence (ChlFl) on the same sample. HPI, hours post inoculation.

In this study, we established a methodology for the analysis of disease progression of FHB in detached oat spikelets. In the first stage, five panicles per oat genotype were harvested and inoculated with *Fusarium* conidia by vacuum treatment per batch experiment. From each vacuum-treated panicle, four spikelets were dissected and placed on *in vitro* plates. Altogether, 20 spikelets per oat genotype in a batch were monitored for disease progression by RGB and ChlFl imaging (**Figures 1**, **2**). Images were taken at 0, at 12, and then at every 24 HPI for 10 days. Flowering spikelets that opened during the time series were removed from the analysis. Spikelets that were not infected despite the treatment or dried out before the end of the observation period were removed from the analysis. In total, time series data from 40 spikelets per oat genotype treatment were collected from the two batches. The extracted data from the two batches were statistically analyzed as combined.

### 3.1 Visual assessment of the disease progression on RGB images

**Figure 3:**
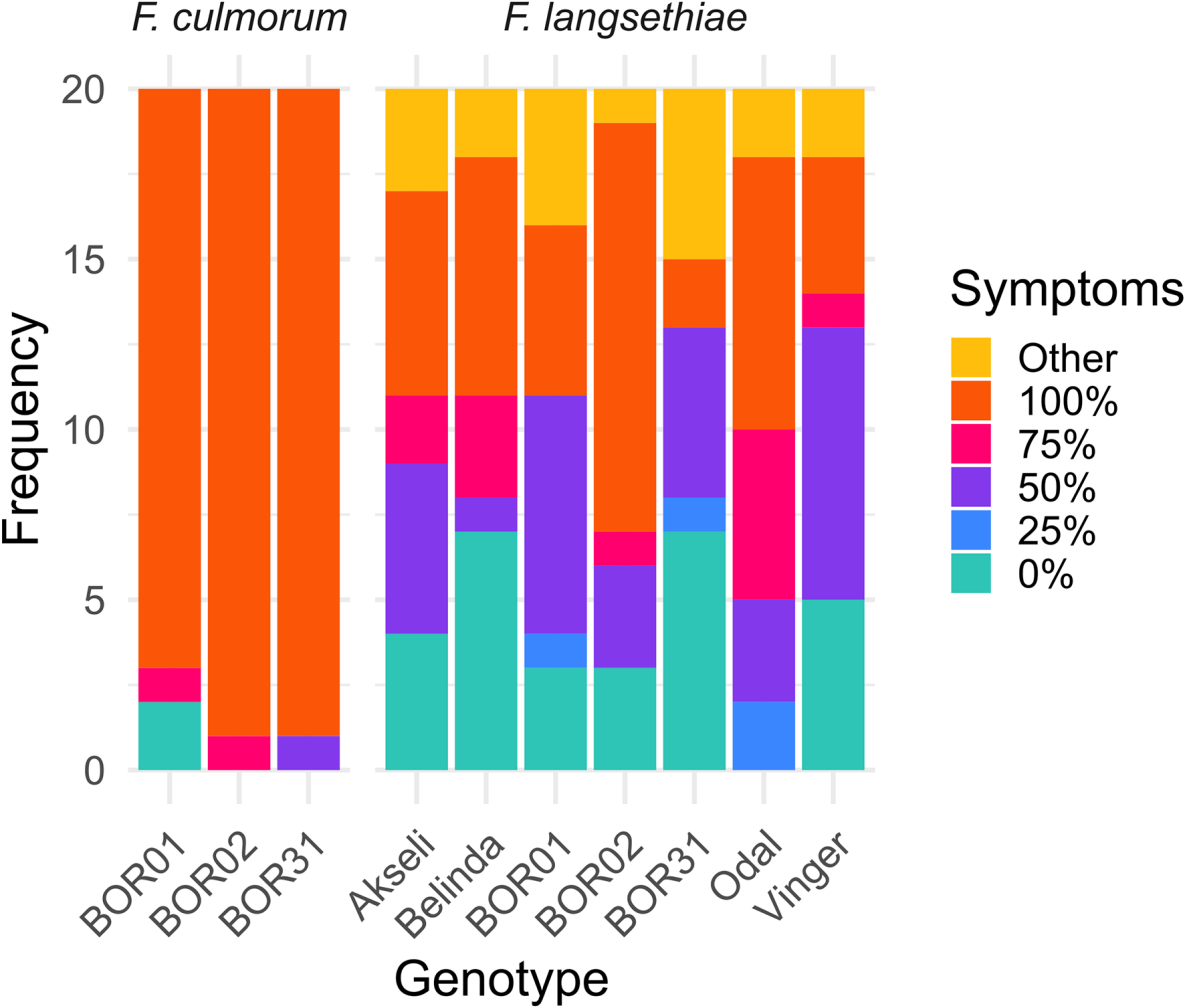
Visual assessment of infection incidence and symptoms on the spikelets by the different treatments at 7 days post infection. The frequencies of spikelets with different percentage of symptomatic tissue are shown as stacked bar plots. The symptomatic percentage was assessed qualitatively by visual scoring and grouped as 0%, 25%, 50%, 75%, and 100% symptomatic tissue, and other damage (yellowing, dark spots). This analysis represents the visual scoring of 20 spikelets per genotype per treatment from batch 2.

RGB images were utilized to qualitatively assess the percentage of spikelet covered by mycelia growth (**Figure 3**). Browning, chlorotic tissue covered with white mycelium was considered as symptomatic tissue. Observations of visible symptoms indicated that *F. langsethiae* infection progressed more slowly (**Figure 2A**) than *F. culmorum* (**Figure 2B**). The incidence and level of visual symptoms in the inoculated spikelets were visually estimated from batch 2 (**Figure 3**), where different responses were observed across cultivars. From the inspected spikelets, only two inoculated with *F. culmorum* were not symptomatic, whereas all others became partly or fully covered by mycelium. At the end of the observation period, only Odal cultivar had symptomatic tissue in all spikelets inoculated with *F. langsethiae*. All other cultivars had variable responses regarding both the incidence and the level of observed symptoms (**Figure 3**). At 7 days after inoculation, *F. culmorum* infections had fully covered the spikelets while the *F. langsethiae* infections were less invasive and mycelia coverage ranged from 0% to 100% coverage.

### 3.2 Estimation of decline in spikelet vitality and spreading of infection over time

**Figure 4:**
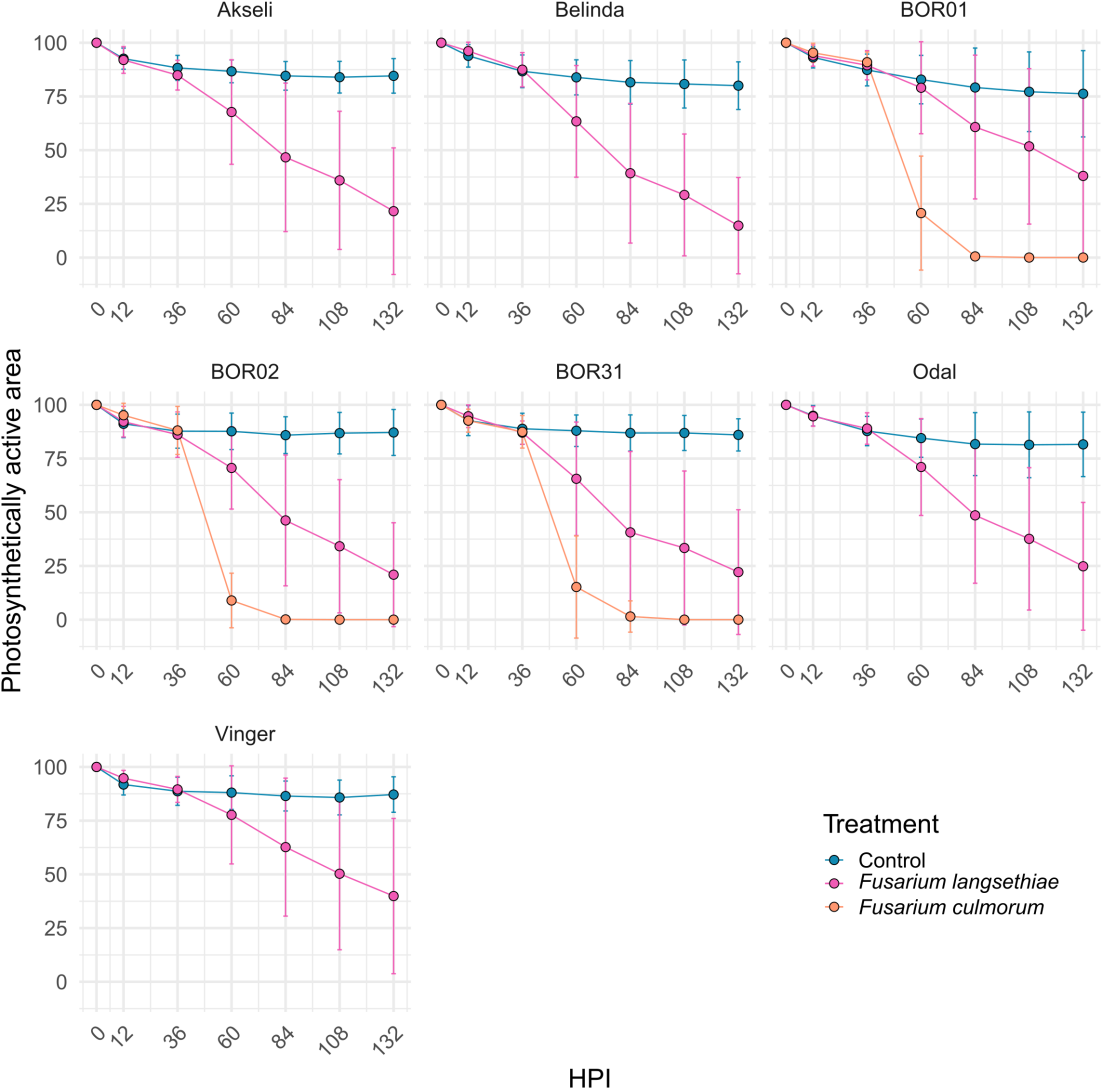
Time series of the photosynthetically active area of spikelets. Each measurement represents the percentage of spikelet fluorescent area relative to its size at 0 hours post inoculation (HPI). All oat genotypes were inoculated with control (mock) and *F. langsethiae*. BOR01, BOR02, and BOR31 were inoculated in addition with *F. culmorum*. Blue line, vacuum control; magenta line, inoculated with *F. langsethiae*; orange line, inoculated with *F. culmorum*. Dots represent means per treatment at each HPI measured and error bars represent standard deviation. This analysis includes combined data from two experimental batches. Each data point represents the mean of 21–38 spikelets.

**Figure 5:**
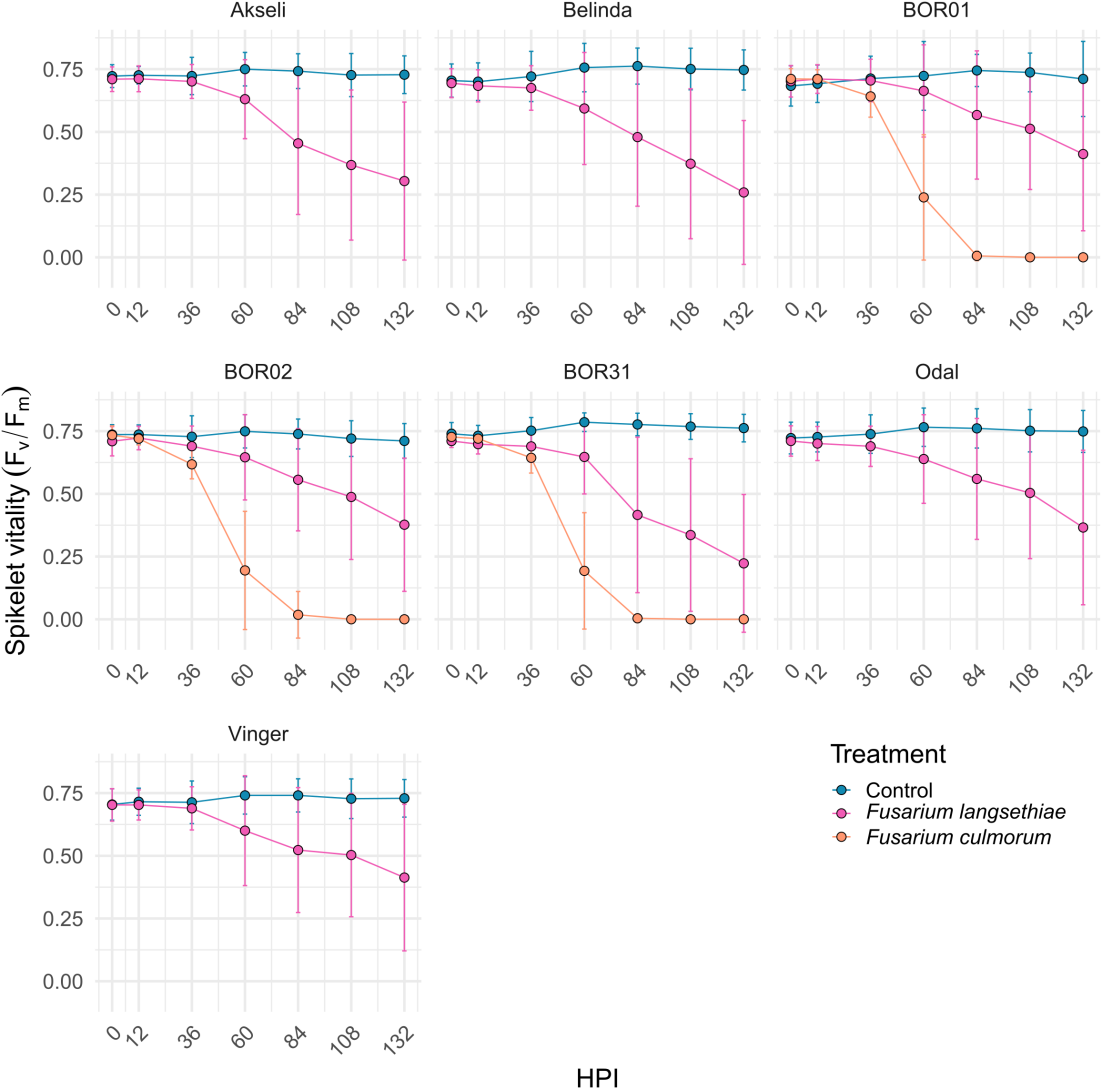
Time series of oat spikelet vitality measured by F_v_/F_m_ mean values per spikelet after *Fusarium* spp. or control inoculation. Data are presented as mean of F_v_/F_m_ values of all fluorescent pixels per spikelet at different hours post inoculation (HPI). All oat genotypes were inoculated with control (mock) and *F. langsethiae*, and BOR01, BOR02, and BOR31 were inoculated in addition with *F. culmorum*. Blue line, vacuum control; magenta line, inoculated with *F. langsethiae*; orange line, inoculated with *F. culmorum*. Dots represent means per treatment at each HPI measured and error bars represent standard deviation. This analysis includes combined data from two experimental batches. Each data point represents the mean of 21–38 spikelets.

Time series analysis of ChlFl images of infected oat spikelets was conducted to quantify the differences in spikelet vitality and infection spreading (**Figure 2**). ChlFl data were collected for each pixel of the spikelets utilizing a scripted macro modified from Pavicic et al. (2021). This strategy allowed the creation of two phenotypes to track disease progression. During the time course of *Fusarium* infection, some spikelet pixels become photosynthetically inactive (loss of ChlFl), or the hyphal growth blocks the ChlFl. Thus, the first phenotype tracked the disappearance of fluorescent pixels over the time course expressed as percentage of the spikelet size at 0 HPI (**Figure 4**). The second phenotype monitored the vitality of the spikelet by averaging F_v_/F_m_ values of fluorescent pixels remaining at each HPI (**Figure 5**). The vacuum inoculation strategy impacted the vitality of the spikelets at early HPI as shown in the control line in **Figure 5**. A recovery from the vacuum inoculation was observed in control spikelets at approximately 60 HPI, which decayed in late HPI likely due to the spikelets being detached from the plant.

The impact of *F. culmorum* to spikelet vitality (mean of F_v_/F_m_, **Figure 5**) over time was very similar for all three oat genotypes. *F. culmorum* evidently separated from the control at early HPI, being significantly different from control treatment at 36 HPI and reaching a value of 0 at 84 HPI (**Supplementary Figure 1**). Mean F_v_/F_m_ values of spikelets inoculated with *F. culmorum* were also clearly distinct from those from *F. langsethiae* inoculation (**Figure 3**) that started declining significantly from the mock-inoculated control between 36 and 60 HPI (**Supplementary Figure 1**). On average, the spikelet vitality of the *F. langsethiae* inoculations remained clearly above 0 for F_v_/F_m_ during the entire analysis period. *F. langsethiae* inoculated spikelets of the breeding lines BOR01 and Vinger became significantly different from the control later (at 84 HPI) than the other oat genotypes, which had clearly declining vitality already at 60 HPI (**Supplementary Figure 1**).

The spreading of fungal infection was illustrated by the relative size of spikelet visible area. In **Figure 4**, the differences between both *Fusarium* inoculation treatments could only be distinguished after 36 HPI (**Figure 4** and **Supplementary Figure 1**). Similar to spikelet vitality, the *F. culmorum* inoculated genotypes showed a rapid decrease in fluorescent spikelet area (declining to 0 from nearly healthy values within 2 days after 36 HPI). This suggests that the fungus was able to cover the whole spikelets, preventing the detection of ChlFl. Contrastingly, the fluorescent area of spikelets inoculated with *F. langsethiae* conidia resembled the behavior of F_v_/F_m_ values at early HPI. However, the time points when the curves presenting the inoculated treatments started to differ from the controls were not identical for different genotypes (**Supplementary Figure 1**) and the shapes of the curves started to look different for different oat genotypes from 60 HPI onwards (**Figure 4**). BOR01 was again the most resistant genotype separating from the control only at 84 HPI (**Figure 4** and **Supplementary Figure 1**). Vinger showed a similar trend and was statistically significant already at 60 HPI, barely reaching the statistical threshold of *P* value < 0.05.

### 3.3 Area under the disease progression curve

**Figure 6:**
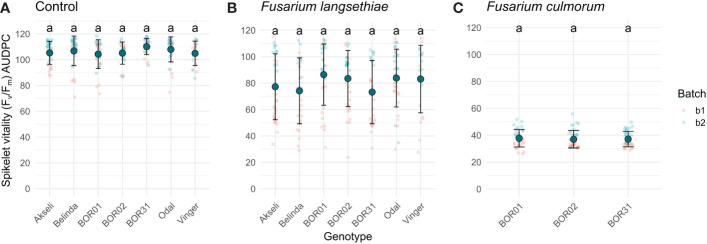
Area under the disease progression curve (AUDPC) of spikelet vitality measured by F_v_/F_m_. Plots show F_v_/F_m_ AUDPC of the seven oat genotypes inoculated with **(A)** mock control, **(B)** *F. langsethiae*, or **(C)** *F. culmorum*. Small red dots correspond to data from batch 1 (b1) and small blue dots correspond to data from batch 2 (b2). Big teal dots represent AUDPC mean, and error bars represent standard deviation. Groups sharing the same letter have no statistical differences at *p* < 0.05 assessed by Tukey HSD *post hoc* test. This analysis includes combined data from two experimental batches. Each data point represents the mean of 21–38 spikelets.

**Figure 7:**
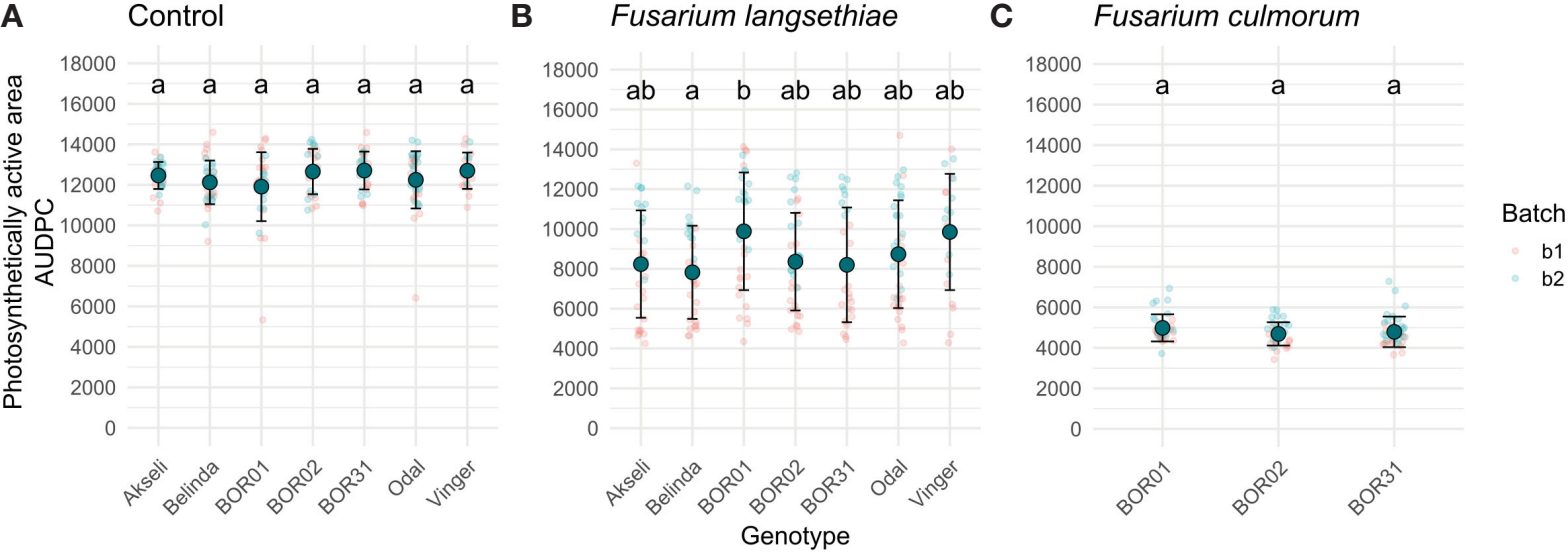
Area under disease progression curve (AUDPC) of spikelet photosynthetic area relative to its size at 0 hours post infection (HPI). Plots show data of the seven oat genotypes used in this study inoculated with **(A)** mock control, **(B)** *F. langsethiae*, or **(C)** *F. culmorum*. Small red dots correspond to data from batch 1 (b1) and small blue dots correspond to data from batch 2 (b2). Big teal dots represent AUDPC mean, and error bars represent standard deviation. Groups sharing the same letter have no statistical differences at *p* < 0.05 assessed by Tukey HSD *post hoc* test. This analysis includes combined data from two experimental batches. Each data point represents the mean of 21–38 spikelets.

To assess differences between oat genotypes in each treatment, the AUDPC was calculated using the method described in Simko and Piepho (2012). This method allows the conversion of an entire time series to a single value that can be assessed with traditional statistical approaches. Thereby, the AUDPC was calculated for each spikelet for both phenotypes used in this study (**Figures 6**, **7**). Although the shapes of the curves representing *F. langsethiae* and the decline in spikelet vitality of different oat genotypes (**Figure 5**) are not identical, the AUDPCs are not significantly different between genotypes (**Figure 6B**), suggesting that, on average, the impact of *F. langsethiae* on spikelet vitality does not differ among the studied oat genotypes. Similar results were observed in *F. culmorum* infections, where no statistical differences in AUDPC were observed between oat genotypes (**Figure 6C**).

The AUDPC of spikelet fluorescent area was significantly larger for genotype BOR01 than for genotype Belinda, indicating the significantly slower spread of infection (**Figure 7B**). Comparison with other oat genotypes yielded no statistical differences in their mean AUDPCs for fluorescent spikelet area. Although not statistically significant, a trend was observed for Vinger cultivar, which had a mean AUDPC closer to BOR1 compared to others having mean values closer to Belinda (**Figure 7B**). No statistical differences were found for *F. culmorum* infections (**Figure 7C**).

## 4 Discussion

### 4.1 Establishing an image-based system for the time series analysis of fungal diseases

FHB is a global threat to cereal grain production especially in humid climates. The disease progression is relatively quick after infecting flowers and heads of cereals and therefore timely detection of the disease would allow early interventions and prevention of further spread of the disease. Thermal and hyperspectral signatures can be utilized as proxies for disease symptoms in field settings. In this study, we established a method to study the FHB disease progression using ChlFl imaging that enables the detection of symptomatic tissues before they are visible to the human eye. When disease progression in individual spikelets is observed both from the RGB images and fluorescent images, we can find at least two advantages compared to previous manually made microscope observations of *Fusarium* infection in oats. First, we can quantify the severity of the infection within the floral tissue, which is important since Tekle et al. (2012) have shown that there can be high variation in the spreading of infections between individual spikelets. The aim was also to develop a system to describe spatial and temporal changes during the disease progression. The recorded RGB images allowed ranking the coverage of hyphae and showed the different rates and efficiencies between the two *Fusarium* species. The ChlFl parameters allowed establishing the decay rate of the healthy tissue as an inverse readout of the disease progression and to detect the timepoint where the oat tissue loses its photosynthetic activity, which may again be difficult to determine by human eye. To allow collecting such data from several oat genotypes treated with two *Fusarium* species, an automated workflow from image capturing to data analysis was established. The temporal analysis was facilitated by the use of automated pixel-specific ChlFl data extraction using the FIJI scripting. Data collection in a time series allowed recording temporal differences between *Fusarium* species in their rate of disease progression and severity, as well as the response time of oat genotypes. Dissecting the disease progression into a time scale will hopefully allow targeting the underlying molecular processes.

### 4.2 Spikelet dissection method allowed observations of disease progression at the organ level

We monitored several different traits, i.e., phenotypes, that can explain the interaction between the selected pathogens and the host plant tissue. These phenotypes included visually estimated disease incidence and the level of visible symptoms, decline of ChlFl-based spikelet vitality (**Figure 5**) over time, and spread of infection based on the decrease of photosynthetically active spikelet area (**Figure 4**) over time. Each of these partially explains what is happening in the interaction between plant genotypes with variable resistance and pathogens with variable aggressiveness. The resistance against FHB causing fungi is described as a complex trait in cereals (Mesterházy et al., 1999) and in oats (Hautsalo et al., 2018). The result of FHB infection in cereals such as mycotoxin contamination in the grain is merely the end outcome from numerous events such as establishment of initial infection in different spikelets (number of infected grain), the spread of infection (severity of infection within a cereal head or in the case of oats within a single spikelet), and the degradation of or tolerance against mycotoxins.

Considering the proportion of infected spikelets, the vacuum inoculation method efficiently infected *F. culmorum* into oat spikelets since only few spikelets had no symptoms after inoculation including in BOR01 genotype that was previously shown as moderately resistant against *Fusarium* infection (Hautsalo et al., 2020). For *F. langsethiae*, the vacuum inoculation method left ~17% of spikelets symptomless, which suggests that this pathogen was not able to infect the spikelets as efficiently. However, *F. langsethiae* is generally considered as a weak pathogen that does not easily cause symptoms (Divon et al., 2012). The vacuum infiltration has been used by Divon et al. (2012) to inoculate oat seeds with *F. langsethiae*, leading to a loss of germination from approximately half of the tested seeds. Nevertheless, compared to yield analyses made from field and greenhouse trials that are inoculated either by sprayings or by sporulation from spawn inoculum (Hautsalo et al., 2018), the non-destructive monitoring of infection over time in vacuum-inoculated spikelets provides novel insight into the plant–disease interaction. With the method described in this study, the individual spikelets are more likely infected, and we can see when and how long the fungi and the plants are interacting.

Based on the findings in this study, it seems that in the case of oats and of a DON-producing fungus such as *F. culmorum*, the number of infected grains might be a critical determinant of infection since we could not find significant differences among genotypes after the infection had been established. This finding is supported by the findings of Hautsalo et al. (2021) where the spikelets from two contrastingly resistant oat genotypes were infected with *F. graminearum* by point inoculation technique but showed no differences at 6 dpi. This type of resistance against the initial infection can be contributed by various mechanisms such as physiological and chemical properties of the flower as well as pathogen-triggered immunity reactions (Hautsalo et al., 2018). However, monitoring of *F. langsethiae* suggests that both the incidence of initial infections and the speed of the spreading of infection might play a role. RNA sampling from the inoculated oats could reveal interesting molecular interactions taking place in the early phases of infection either between 0 and 36 HPI when no difference in the phenotype between the inoculated and the control spikelets can be seen or as far as 84 HPI when no photosynthesis can be detected for *F. culmorum* and respectively from 12 to 132 HPI or even longer for *F. langsethiae.* Nevertheless, the final ranking of the genotypes out in the field might still be attributed to other factors such as the evasion provided by the differences in flowering habit (Herrmann et al., 2020).

### 4.3 Aggressiveness of different *Fusarium* species

The findings related to the infection of two different *Fusarium* species in our study are in accordance with the literature. Divon et al. (2019) observed the *F. langsethiae* to have a similar infection pathway and very similar infection structures to *F. graminearum*, but the rate of spreading of *F. langsethiae* infection was shown to be up to three times slower. Divon also mentioned to have observed that the spores of *F. langsethiae* germinate 10 to 15 h later than the spores of *F. graminearum.* The DON-mycotoxin-producing species, *F. graminearum* and *F. culmorum*, are closely related and have resemblance in their infection pathway. Kang and Buchenauer (2000) observed that *F. culmorum* spores germinated and formed more than one germ tube inside a wheat spikelet. At the beginning of the infection, they also observed hyphal networks forming on the internal surfaces of the spikelet, and by 36 HPI, the ovary and stigma were also infected. Tekle et al. (2012) observed the growth of *F. graminearum* in oat spikelet through a microscope and found that hyphal growth was observed as early as 1 DPI and the entire florets were often colonized at 3 DPI, whereas for *F. langsethiae*, up to 14 days was needed to detect colonization of all surfaces of the spikelet (Divon et al., 2019). In our study, we start to observe damage in the photosynthetic apparatus during the second day after inoculation in the case of both fungal species, but we did not observe a sharp decline in spikelet vitality in *F. langsethiae* as we did for *F. culmorum* at any point during the entire observation period. This suggests that a longer observation period might be needed to show how low the mean spikelet vitality would decline or how large proportion of the spikelets may eventually become covered by *F. langsethiae*. Unfortunately, a longer observation period would also start to harm the control spikelets as the plates start to dry out.

Considering that the *Fusarium* spp. in this study are hemibiotrophic fungi (Wagacha and Muthomi, 2007; Walter et al., 2010) might help to understand what happens during the time series of our study. The length of symptomless phase in our data is similar to the one reported for wheat, where 24 to 32 h are the expected duration of the parasitic phase before shifting to saprophytic phase (Gottwald et al., 2012). In this last phase, dead plant tissue is consumed and killed by the fungus, producing loss of ChlFl and visible degradation of the spikelet. The parasitic phase of *F. langsethiae* appears to be relatively longer. However, the complete coverage for some spikelets by hyphae is a sign that *F. langsethiae* can also effectively derive nutrients from dead plant tissue and spread efficiently. This phenomenon was observed in this study in the vitality (F_v_/F_m_) of some spikelets reaching zero by the end of the observation period.

The progression of infection is clearly faster for the DON-producing *F. culmorum* than it is for *F. langsethiae*, which does not produce DON mycotoxin. In wheat literature, an *F. graminearum* strain without functional gene for DON production was clearly less aggressive in disease development (Boddu et al., 2007). Wheat resistance against *F. graminearum* depends on the activation at the right time of several defense pathways regulated by phytohormones (Glazebrook, 2005) that work against either parasitic or necrotrophic pathogens (Ding et al., 2011). Diamond et al. (2013) found DON to inhibit programmed cell death in *Arabidopsis* cell cultures, and they suggested that low levels of DON may facilitate pathogen establishment in the initial biotrophic phase of *Fusarium* infection whereas a higher level of DON may support the necrotrophic phase. Tan et al. (2020) also discovered from wheat that pre-inoculation of *F. poae* reduced the abundance of later inoculated *F. graminearum* when compared to *F. graminearum* inoculated alone. This indicates that a defense response activated by a *Fusarium* species that does not produce DON can still provide shelter from a DON-producing fungus. This finding suggests that universal resistance mechanisms against FHB pathogens could exist. Further studies should focus on the complex interactions between hosts and several fungal pathogens, and the phenotyping methods described in this study can be suggested for such studies.

### 4.4 Resistance and susceptibility in oat cultivars

The oat varieties in this study represented the known variation in resistance against both *Fusarium* species used in inoculations. Recently published findings from naturally infected experiments made in Norway over a 10-year period (Hofgaard et al., 2022) describe several differences in our genotypes well. Cultivar Odal is expected to contain resistance against the DON-producing fungi but lacks resistance to *F. langsethiae* and cultivar Vinger is expected to be moderately resistant against both fungi. Cultivar Belinda was found to be susceptible to both *F. langsethiae* infection and DON producers, while cultivar Odal has been found to be even more susceptible. In another study (Hautsalo et al., 2020), cultivar BOR31 was highly susceptible to infections and DON accumulation in greenhouse, whereas cultivar Akseli was moderately resistant to infection. The same study showed that the breeding line BOR01 was the best line tested in greenhouse conditions having both a low level of DON accumulation and infected kernels. Belinda was also included in these experiments, and it had a relatively high number of infected kernels. Line BOR02 was selected based on its promising results in a low number of unpublished experiments.

The only difference found in *F. culmorum*-infected genotypes was that a couple of spikelets from BOR01 were not infected whereas spikelets of all other genotypes were infected, suggesting that BOR01 has resistance against the initial infection. This effect was observed in neither spikelet vitality nor the photosynthetically active area, where all three genotypes infected with *F. culmorum* showed no statistical difference and they separated from the control treatment together at 60 HPI. Similar observations were found by analyzing the AUDPC for both phenotypes. Interestingly, some differences were observed before 60 HPI, but we hypothesized that they were due to vacuum inoculation rather than *F. culmorum* infection. Having more time points in the analysis might also help reveal different progression patterns on different oat genotypes in future studies.

In *F. langsethiae* inoculations, only BOR01 and Vinger showed some resistance to the disease progression. Mostly all remaining genotypes separated from the control treatment at 60 HPI for both phenotypes. The exceptions were Akseli and BOR31 that showed a modest susceptibility already at 36 HPI for the photosynthetically active area and vitality, respectively. The analysis of AUDPC only showed statistical differences between the susceptible cultivar Belinda and BOR01 genotypes in the photosynthetically active area. Furthermore, Vinger genotype was not statistically significant from the other lines in the photosynthetically active area but, on average, was one of the most resistant. This result suggests that the analysis of each time point was superior to the analysis of time course trajectories to find subtle differences between genotypes. Interestingly, the cultivar Odal does not show susceptibility here, but maybe the high T-2/HT-2 contaminations in field (Hofgaard et al., 2022) are due to the higher proportion of infected kernels within the samples. Further studies are necessary to determine which mechanisms interact behind the found phenotypical differences and trends.

## 5 Conclusions

Improving the tolerance of cereals and especially oats against the FHB disease is a long journey. The resistance against FHB is known to be complex and impacted by several minor genes. Only a few quantitative trait loci (QTL) are currently determined against FHB in oats (Bjørnstad et al., 2017, Isidro-Sánchez et al., 2020). In contrast to previous genetic studies that are based on heavy field phenotyping, the phenotyping method presented in this study provides information from the specific plant tissue. The experiments presented here were executed under controlled environment and infection conditions, preventing environmental disturbances occurring as in field trials. With these controlled phenotyping methods, we are one step closer to discovering the small differences behind the resistance. To allow developing time series analysis of the disease progression, dissected spikelets were imaged after inoculation treatments. Two different *Fusarium* spp. were utilized to obtain differential responses and an image-based method to allow recording the responses in space and time. We believe that this method will pave the way for detailed characterization of the disease progression in susceptible and resistant oat varieties. Rather than engaging this spikelet dissection method for breeding selection of oat cultivars for variable resistance, we propose this method for the identification of molecular markers specific for the different stages of FHB disease progression. Controlled experiments play a critical role in the molecular characterization of biological processes. A biological process can be standardized in a time series setup that allows defining samples for molecular analysis and thereby discoveries of molecular markers. We also presented here two new oat phenotypes that could be used for the discovery of molecular markers in genome-wide association studies.

## Data availability statement

The original contributions presented in the study are included in the article/**Supplementary Material**. Further inquiries can be directed to the corresponding author.

## Author contributions

Conceptualization: MP, JH, KH, KM, and MJ; methodology: MP, JH, KM, and KH; software: MP and KM; validation: MP and KM; formal analysis: KM, MP, JH, and KH; investigation: KM and KH; resources: KH, MJ, and DJ; data curation: KM and MP; writing: MP, JH, KH, and KM; visualization: MP; supervision: KH and MJ; project administration: KH, MJ, and DJ; funding acquisition: MJ, KH, DJ, and JH. All authors contributed to the article and approved the submitted version.

## Supplementary material

Supplementary Figure 1 Daily statistical significances of time course analysis. Spikelet vitality **(A)** *F. langsethie* **(B)** *F. culmorum*. Spikelet photosynthetically active area **(C)** *F. langsethiae* and **(D)** *F. culmorum*. Dotted line adjusted *P* value = 0.05.
