# Supplementary figures and images for: Image-based time series analysis to establish differential disease progression for two Fusarium head blight pathogens in oat spikelets with variable resistance
Source: Front Plant Sci. 2023 Mar 14;14:1126717. doi: 10.3389/fpls.2023.1126717 (PMC10043315; doi:10.3389/fpls.2023.1126717)

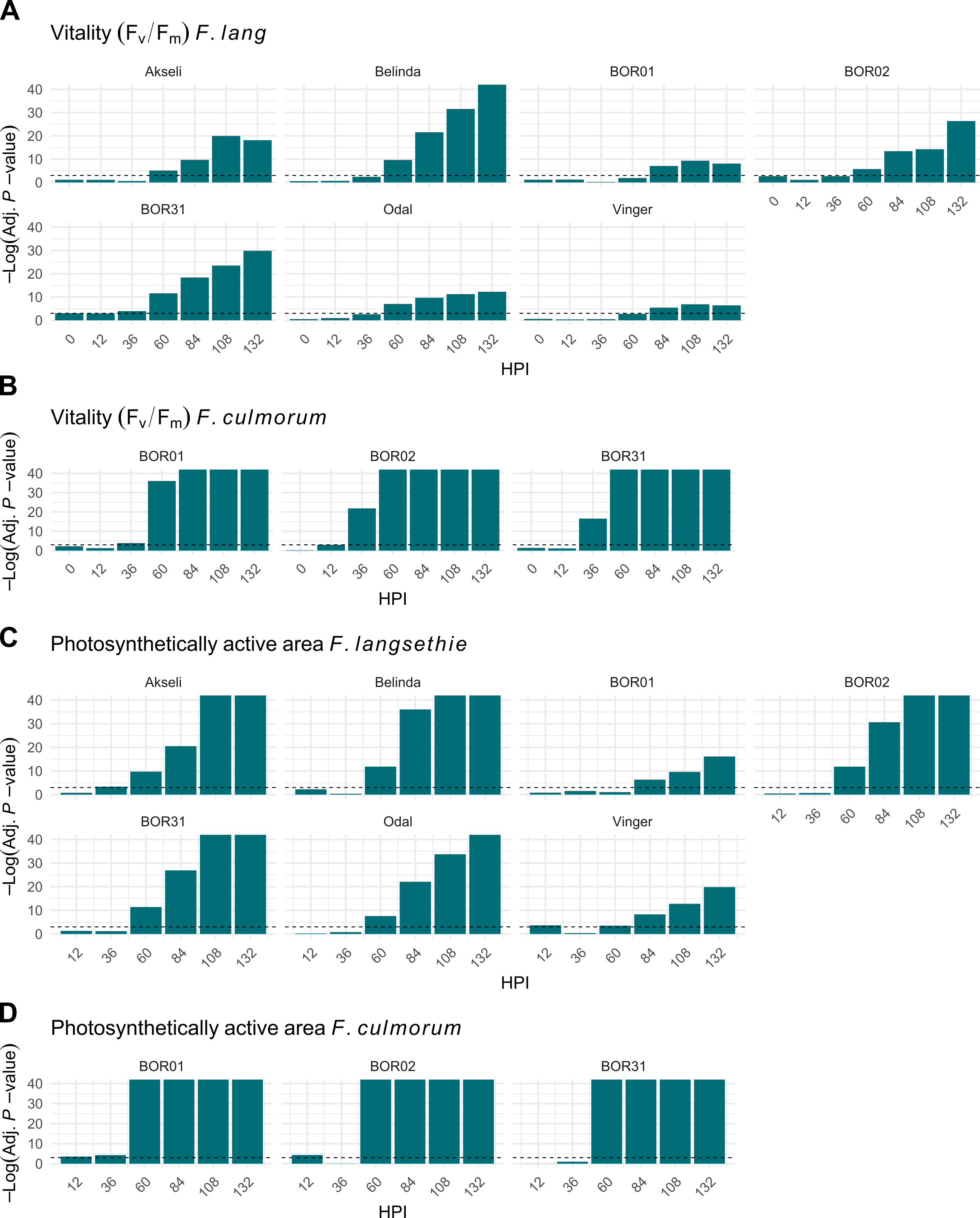

Supplement: Supplementary Figure 1 — Daily statistical significances of time course analysis. Spikelet vitality (A) F. langsethie (B) F. culmorum. Spikelet photosynthetically active area (C) F. langsethiae and (D) F. culmorum. Dotted line adjusted P value = 0.05. [file Image_1.tiff]
